# Anterior urethra sparing cystoprostatectomy for bladder cancer: a 10-year, single center experience

**DOI:** 10.1186/s40064-015-1200-7

**Published:** 2015-08-08

**Authors:** Nozomi Hayakawa, Nobuyuki Kikuno, Hiroki Ishihara, Osamu Ryoji, Kazunari Tanabe

**Affiliations:** Department of Urology, Saiseikai Kawaguchi General Hospital, Nishikawaguchi, Kawaguchi, Saitama Japan; Department of Urology, Tokyo Women’s Medical University, Shinjyuku-ku, Tokyo, Japan

**Keywords:** Bladder cancer, Cystectomy, Urethra, Urinary diversion

## Abstract

**Purpose:**

Decision making regarding the urethra before and after radical cystectomy due to urothelial carcinoma has always been controversial. To determine whether anterior urethra sparing cystoprostatectomy for bladder cancer is an oncologically-safe procedure, we evaluated the long-term oncologic clinical outcome.

**Patients and methods:**

A total of 51 male patients with cTa-4N0-2M0 bladder cancer were treated with anterior urethra sparing cystoprostatectomy and simultaneous urinary diversion between 2000 and 2013, and underwent follow up for 4 months or more. We assessed differences in the perioperative outcomes, oncologic outcomes and recurrence rates according to the urinary diversion.

**Results:**

The median patient age and follow-up period were 66 years and 35 months, respectively. The 5- and 10-year recurrence free survival (RFS) rates in ileal conduit (IC) group vs. orthotopic neobladder reconstruction (NB) group were 45.0 and 20.3% vs. 39.3 and 19.6%, respectively. Likewise, the 5- and 10-year disease specific survival (DSS) were 52.7 and 32.1% vs. 39.3 and 29.5%, respectively. Multivariate analysis revealed two independent prognostic factors for RFS and DSS, including age at surgery and lymph node status. Local recurrence in the remnant anterior urethra occurred in only 1 patient (2.0%) at 57 months after surgery.

**Conclusions:**

Our long-term data show that anterior urethra sparing cystoprostatectomy is an oncologically-safe procedure regardless of the type of urinary diversion in a subset of carefully selected patients with bladder cancer without evidence of urothelial carcinoma in the urethra/bladder neck and urethral surgical margin.

**Electronic supplementary material:**

The online version of this article (doi:10.1186/s40064-015-1200-7) contains supplementary material, which is available to authorized users.

## Background

Radical cystoprostatectomy is the standard treatment in men with muscle-invasive bladder cancer (MIBC) or refractory high risk non-MIBC (Gakis et al. 2013; Stenzl et al. 2011). This includes removal of the bladder, prostate, seminal vesicles and *vasa deferentia* (Gakis et al. 2013). The overall incidence of urethral recurrence (UR) in contemporary cystectomy patients is 4–6%. In patients undergoing orthotopic bladder replacement, the incidence is lower (Huguet 2013; Huguet et al. 2008; Huguet 2012; Stein et al. 2005; Boorjian et al. 2011). The most consistent risk factor associated with the occurrence of UR is the involvement with urothelial prostate tumor, and especially, prostatic stromal involvement (Stein et al. 2005; Nieder et al. 2004; Hardeman and Soloway 1990).

Perineal urethrectomy is indicated with cystectomy in male patients in the presence of urethral involvement by cancer or if there is a risk of urethral reinvolvement, such as a positive prostatic urethral margin, prostatic urethral superficial urothelial carcinoma (UC) or prostatic stromal invasion (Cho et al. 2009). It has been also reported to be a more complicating procedure as a result of the additional perineal wound, longer operation duration and delayed ambulation (Elshal et al. 2011). Therefore, it remains a controversial matter whether simultaneous prophylactic urethrectomy or urinary diversion with radical cystoprostatectomy should be performed even for cases in which the principal risk factors for UR have been identified preoperatively. Moreover, there are few reports of remnant urethral recurrence in male patients undergoing ileal conduit with no prophylactic urethrectomy.

We present our experience in evaluating whether anterior urethra sparing cystoprostatectomy for bladder cancer influences into the incidence of remnant urethral recurrence and subsequent oncologic clinical outcome, based upon differences of simultaneous urinary diversion.

## Results and discussion

### Clinical presentation, diagnosis, and pathological findings

Ultimately, a study population of 51 males with cTa-4N0-2M0 bladder cancer undergoing anterior urethra sparing cystoprostatectomy and simultaneous urinary diversion was eligible for analysis. The patient characteristics are summarized in Additional file 1: Table S1. The median age of the patients was 66 years (range 48–84 years). Of the 56 consecutive patients, 5 patients were excluded for the following reasons: 2 patients had no urinary diversion caused by chronic renal failure requiring maintenance hemodialysis among their comorbid disorders, 2 patients had undergone percutaneous ureterostomy as urinary diversion and bone metastasis, and the another one patient was diagnosed as having squamous cell carcinoma post-pathologically. IC and NB were selected as the approach to urinary diversion in 25 (49.0%) and 26 (51.0%) patients, respectively. The median follow up time was 35 months (range 4–143 months). Upon review of the final cystoprostatectomy specimen, the presence of carcinoma in situ (CIS), non-organ confined disease (pT3 or greater), and node-positive disease were evident in 10 (19.6%), 19 (37.3%), and 9 (17.6%) patients, respectively. A total of four patients (7.9%) demonstrated prostatic urethral involvement. While one patient had pathological prostatic ductal disease, three exhibited stromal involvement. There were no differences in the presence of CIS, pathological stage, UC grade, and prostate involvement when comparing patients undergoing IC vs. NB urinary diversion.

### Perioperative outcomes and complications

The median operation time was significantly shorter for the IC group (546 min) compared with the NB group (594 min) (*P* = 0.027), However, there were no statistical differences in the median blood loss or the number of hospitalization days between these subgroups (1,500 vs. 1,700 ml and 47 vs. 41 days in the IC group vs. NB group, respectively) (Additional file 2: Table S2). The surgical margins were tumor free for all cases.

While there was no perioperative mortality, a total of 19 (37.3%) patients experienced perioperative complications. Both ileus and pyelonephritis occurred as the most frequent complication in 4 (7.8%) patients each. There was no significant impact on the complication rate and type between these subgroups (Additional file 3: Table S3).

### Oncologic outcomes and recurrence rates according to the site in patients with postoperative recurrence

**Fig. 1:**
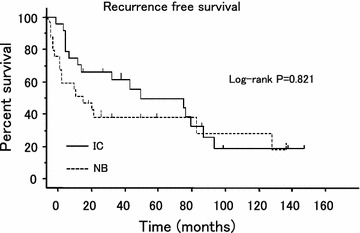
Actual recurrence free survival in 51 patients stratified by the type of urinary diversion.

**Fig. 2:**
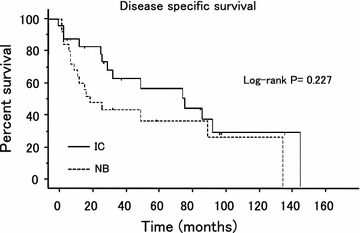
Actual disease specific survival in 51 patients stratified by the type of urinary diversion.

Five- and 10-year RFS by Kaplan–Meier estimation in IC group vs. NB group were 45.0 and 20.3% vs. 39.3 and 19.6%, respectively (p > 0.05) (Fig. 1). Similarly, the 5- and 10-year DSS were 52.7 and 32.1% vs. 39.3 and 29.5%, respectively (p > 0.05) (Fig. 2). Multivariate analysis revealed three independent prognostic factors for RFS, including age at surgery (p = 0.03), maximum pathological tumor stage (p = 0.01), lymph node status (p = 0.0001) and two independent prognostic factors for DSS, including age at surgery (p = 0.006), lymph node status (p = 0.03) (Additional file 4: Table S4). None of the other factors tested proved to be of prognostic significance. Patients with lymph node-positive disease or who were over 60 years old demonstrated significantly worse survival and higher recurrence rates than those with no lymph node involvement or who were under 60 years of age (p = 0.013 and 0.001 or p = 0.001 and 0.001, respectively). Recurrence developed during follow-up in 20 of the 51 patients (39.2%), including distant recurrence of abdominopelvic lymph node, lung, bone, liver and local recurrence of muscle, rectum, ureter and remnant urethra (Additional file 5: Table S5). A total of 9 patients (17.6%), comprised of 6 in the IC group and 3 in the NB group presented with distant, as well as local recurrences. Remnant urethral recurrence was identified in only one patient in the NB group (2.0%) at 57 postoperative months, although other recurrence sites were more frequently confirmed during the same follow-up period. There were no significant differences in the recurrence rates according to the site in patients with postoperative recurrence between each subgroup.

Decision making regarding the urethra before and after radical cystectomy due to urothelial carcinoma has always been controversial (Huguet 2011). The incidence of urethral recurrence of UC after radical cystectomy has been estimated to be as high as 10% (Freeman et al. 1994). Significant attention has been directed toward determining appropriate patient selection for urethral preservation and orthotopic neobladder reconstruction. It is generally accepted that orthotopic diversion should not be performed if there is a high risk of tumor recurrence in the retained urethra. While the pathological features considered as potentially high risk have evolved during the last decade, with some of these factors surgeons continue to consider prophylactic urethrectomy, thereby precluding orthotopic diversion (Hassan et al. 2004). These characteristics include prostatic urethral involvement in men, bladder neck and/or vaginal wall involvement in women and CIS (Freeman et al. 1996). By contrast, there have been few reports in which intraoperative frozen section biopsies of the urethral margin have been used systematically and with sufficient follow-up; although most authors currently agree that this approach would determine whether orthotopic diversion or urethrectomy should be performed (Huguet 2011). Kassouf et al. (2008) reported that a positive preoperative transurethral resection prostatic urethral biopsy did not correlate with final margin and should not exclude patients from consideration for orthotopic urinary diversion in their study of 245 radical cystectomy specimens for UC. There are also a few reports of remnant urethral recurrence in male patients who had undergone the ileal conduit procedure without prophylactic urethrectomy. Based on these reports, we hypothesized that simultaneous prophylactic urethrectomy with radical cystoprostatectomy might not always be necessary, regardless of the type of urinary diversion, if the urethral margin alone was negative on frozen section. In our series, surgical margins were confirmed to be tumor free for all cases. Therefore, prophylactic urethrectomy was not performed and the types of urinary diversion were divided into IC and NB groups, according to the complete assessment of several factors, including patient age, patient desire, preoperative complications, past history of digestive disease, intraoperative findings, such as the length of the mesentery or the presence of some adhesion. As shown in Additional file 1: Table S1, there were no significant differences in the patient background among those patients undergoing IC vs. NB urinary diversion. As a result, the histopathological findings in our retrospective study revealed that 4 patients (7.9%) exhibited prostatic involvement even if the negative urethral surgical margin was confirmed intraoperatively. However, remnant urethral recurrence was identified in only one patient (2.0%) who interestingly exhibited no prostatic involvement.

In recent years, surgeons have begun to report case series of minimally invasive radical cystectomy for the treatment of bladder cancer, including laparoscopic radical cystectomy (LRC) and robot-assisted radical cystectomy (RARC), demonstrating the surgical feasibility of these procedure with the potential of reduced blood loss and more rapid return of bowel function and hospital discharge (Nix et al. 2010; Challacombe et al. 2011). LRC and RARC are increasingly performed; although open radical cystectomy (ORC) remains the standard approach for bladder cancer (Yuh et al. 2015; Stenzl et al. 2011; Snow-Lisy et al. 2014; Bochner et al. 2014; Linder et al. 2014). Furthermore, Tyritzis et al. (2013) reported that outcomes after RARC with totally intracorporeal neobladder diversion appeared satisfactory and consistent with a contemporary open series. Despite these excellent experiences and observations, high levels of clinical evidence with respect to the significance of urethral management, such as LRC or RARC with simultaneous prophylactic urethrectomy, are lacking. Perineal urethrectomy has been reported to be a complicating procedure as a consequence of the extra perineal wound, longer operation duration and delayed ambulation, although radical cysto-urethrectomy is recommended for every patient with bladder cancer who has a urethral tumor or a risk of urethral reinvolvement (Elshal et al. 2011). Under this recent trend, minimally invasive surgical techniques have achieved increasing support and the decision making process regarding the urethra before and after cystectomy due to bladder cancer has become an even more difficult problem.

In our study, patients with lymph node-positive disease or those who were over 60 years old demonstrated significantly worse survival and higher recurrence rates than those with no lymph node involvement or those who were younger than 60 years. However, there were no statistically significant differences in perioperative outcomes, except for the median operation time, oncologic outcomes and recurrence rates based upon the urinary diversion approach. Remnant urethral recurrence was identified in only one patient (2.0%). These results are similar to the previous reports (Taylor et al. 2010; Hautmann et al. 2006). Our data indicate that anterior urethra sparing cystoprostatectomy for bladder cancer did not influence the incidence of remnant urethral recurrence and oncologic clinical outcome based upon different approaches to simultaneous urinary diversion. We believe that our study could be very useful in consideration of the significance of simultaneous prophylactic urethrectomy with radical cystectomy, regardless of LRC, RARC, or ORC.

Our study is limited by the retrospective, non-randomized design and by the small number of patients. A randomized clinical trial focused on the definitive urethral management before and after minimally invasive radical cystectomy will be necessary in the future.

## Conclusions

Our long-term data indicate that anterior urethra sparing cystoprostatectomy is an oncologically-safe procedure, regardless of the type of urinary diversion, in a subset of carefully selected patients with bladder cancer without evidence of urothelial carcinoma in the urethra/bladder neck or the urethral surgical margin.

## Methods

### Study population

We retrospectively reviewed the medical records of 56 consecutive male patients who underwent an anterior urethra sparing cystoprostatectomy for primary bladder cancer at our institute between December 2000 and September 2013, who were included in a computerized database containing comprehensive clinical and pathological data. All female patients were excluded. Patients were excluded from analysis when they did not have pathological evidence of urothelial carcinoma or did not undergo IC or NB. The final pathological analysis was performed using routine step sectioning, which was the protocol during this period. The primary bladder lesion was classified using the TNM classification (Edge et al. 2010).

### Treatment and follow-up

Indication for radical cystectomy were muscle invasive bladder cancer (MIBC) or high-risk non MIBC (NMIBC) including multifocal T1 tumors, T1 tumors located in some difficult sites for complete resection, residual T1 after re-resection, T1 with concomitant CIS, and BCG-refractory tumors. This study cohort had undergone anterior urethra sparing cystoprostatectomy and urinary diversion. Several forms of urinary diversion were selected, as follows: IC, NB and ureterocutaneostomy. Patient follow-up was relatively uniform and included urinary cytology, abdomen-pelvis computed tomography, whole-body bone scan, and chest X-ray. These tests were performed at 3-month intervals for the initial 2 years, at 6-month intervals for the subsequent 3 years, and annually thereafter. Recurrence was confirmed by pathology findings and/or based on imaging findings in all patients classified as exhibiting tumor recurrence. The diagnosis of urethral recurrence was confirmed pathologically in the resected transurethral tissue samples.

### Study methods

Clinical information was obtained by a retrospective review of all patient medical records. Preoperative tumor stage was determined according to the UICC. We investigated T stage, N stage, M stage, tumor grade, concomitant CIS, and urethral surgical margin status. To rule out macroscopic tumor formation in the bladder neck, posterior or anterior urethra, all patients underwent preoperative urethrocystoscopy. Negative urethral surgical margins were confirmed by pathological diagnosis during surgery. The clinical and pathological characteristics of these patients are described in Additional file 1: Table S1.

### Outcomes

We assessed the perioperative outcomes (operation time, blood loss, the number of hospitalization days, perioperative complications), oncologic outcomes, including RFS and DSS, and recurrence rates according to the different approaches to simultaneous urinary diversion. RFS and DSS were defined as the time from anterior urethra sparing cystoprostatectomy with simultaneous urinary diversion until recurrence and the date of death from bladder cancer or last follow up, respectively. Patients were monitored until death or loss to follow-up; the median values are reported for age at the time of operation and the duration of follow-up.

### Statistical analysis

Results were analyzed using descriptive statistical analysis. Survival data were analyzed using Kaplan–Meier survival estimates. The log-rank test was used to compare RFS and DSS in the patient subgroups. Cox proportional hazard regression models were used to evaluate independent prognostic factors associated with RFS and DSS in this patient cohort. Stat mate III for Windows was used for the statistical analyses and two-sided p-values <0.05 were considered significant.

## Additional files

Additional file 1:
**Table S1.** Demographic and pathological characteristics of 51 male patients undergoing anterior urethra sparing cystoprostatectomy and simultaneous urinary diversion for bladder UC. Additional file 2:
**Table S2.** Perioperative outcomes. Additional file 3:
**Table S3.** Perioperative complications. Additional file 4:
**Table S4.** Multivariate analysis of factors associated with RFS and DSS. Additional file 5
**Table S5.** Recurrence rates according to the site in patients with postoperative recurrence.

## Abbreviations

MIBC: muscle-invasive bladder cancer
UR: urethral recurrence
UC: urothelial carcinoma
CIS: carcinoma in situ
IC: ileal conduit
NB: orthotopic neobladder reconstruction
LRC: laparoscopic radical cystectomy
RARC: robot-assisted radical cystectomy
ORC: open radical cystectomy
UICC: Unio Internationalis Contra Cancrum
RFS: recurrence free survival
DSS: disease specific survival
