# Supplementary material for: Anterior urethra sparing cystoprostatectomy for bladder cancer: a 10-year, single center experience
Source: Springerplus. 2015 Aug 8;4:401. doi: 10.1186/s40064-015-1200-7 (PMC4529429; doi:10.1186/s40064-015-1200-7)
Supplement: Additional file 1: — Table S1. Demographic and pathological characteristics of 51 male patients undergoing anterior urethra sparing cystoprostatectomy and simultaneous urinary diversion for bladder UC. [file 40064_2015_1200_MOESM1_ESM.ppt]

## Slide 1
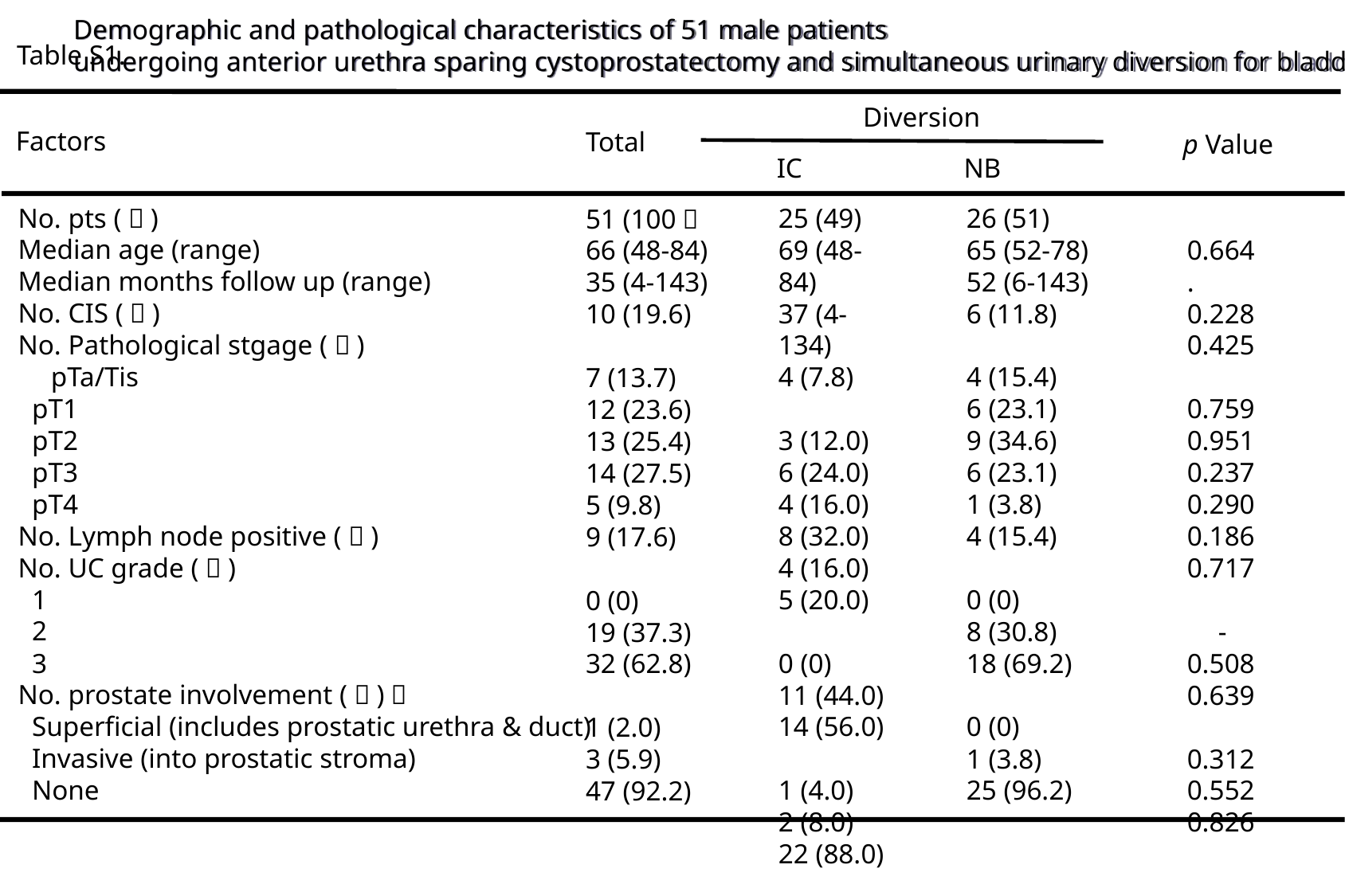

Demographic and pathological characteristics of 51 male patients
undergoing anterior urethra sparing cystoprostatectomy and simultaneous urinary diversion for bladder UC
Table S1.
Diversion
Factors
Total
p Value
IC
NB
No. pts (％)
Median age (range)
Median months follow up (range)
No. CIS (％)
No. Pathological stgage (％)
　pTa/Tis
 pT1
 pT2
 pT3
 pT4
No. Lymph node positive (％)
No. UC grade (％)
 1
 2
 3
No. prostate involvement (％)：
 Superficial (includes prostatic urethra & duct)
 Invasive (into prostatic stroma)
 None
25 (49)
69 (48-84)
37 (4-134)
4 (7.8)
3 (12.0)
6 (24.0)
4 (16.0)
8 (32.0)
4 (16.0)
5 (20.0)
0 (0)
11 (44.0)
14 (56.0)
1 (4.0)
2 (8.0)
22 (88.0)
26 (51)
65 (52-78)
52 (6-143)
6 (11.8)
4 (15.4)
6 (23.1)
9 (34.6)
6 (23.1)
1 (3.8)
4 (15.4)
0 (0)
8 (30.8)
18 (69.2)
0 (0)
1 (3.8)
25 (96.2)
0.664.
0.228
0.425
0.759
0.951
0.237
0.290
0.186
0.717
-
0.508
0.639
0.312
0.552
0.826
51 (100）
66 (48-84)
35 (4-143)
10 (19.6)
7 (13.7)
12 (23.6)
13 (25.4)
14 (27.5)
5 (9.8)
9 (17.6)
0 (0)
19 (37.3)
32 (62.8)
1 (2.0)
3 (5.9)
47 (92.2)
