# Supplementary material for: Anterior urethra sparing cystoprostatectomy for bladder cancer: a 10-year, single center experience
Source: Springerplus. 2015 Aug 8;4:401. doi: 10.1186/s40064-015-1200-7 (PMC4529429; doi:10.1186/s40064-015-1200-7)
Supplement: Additional file 2: — Table S2. Perioperative outcomes. [file 40064_2015_1200_MOESM2_ESM.ppt]

## Slide 1
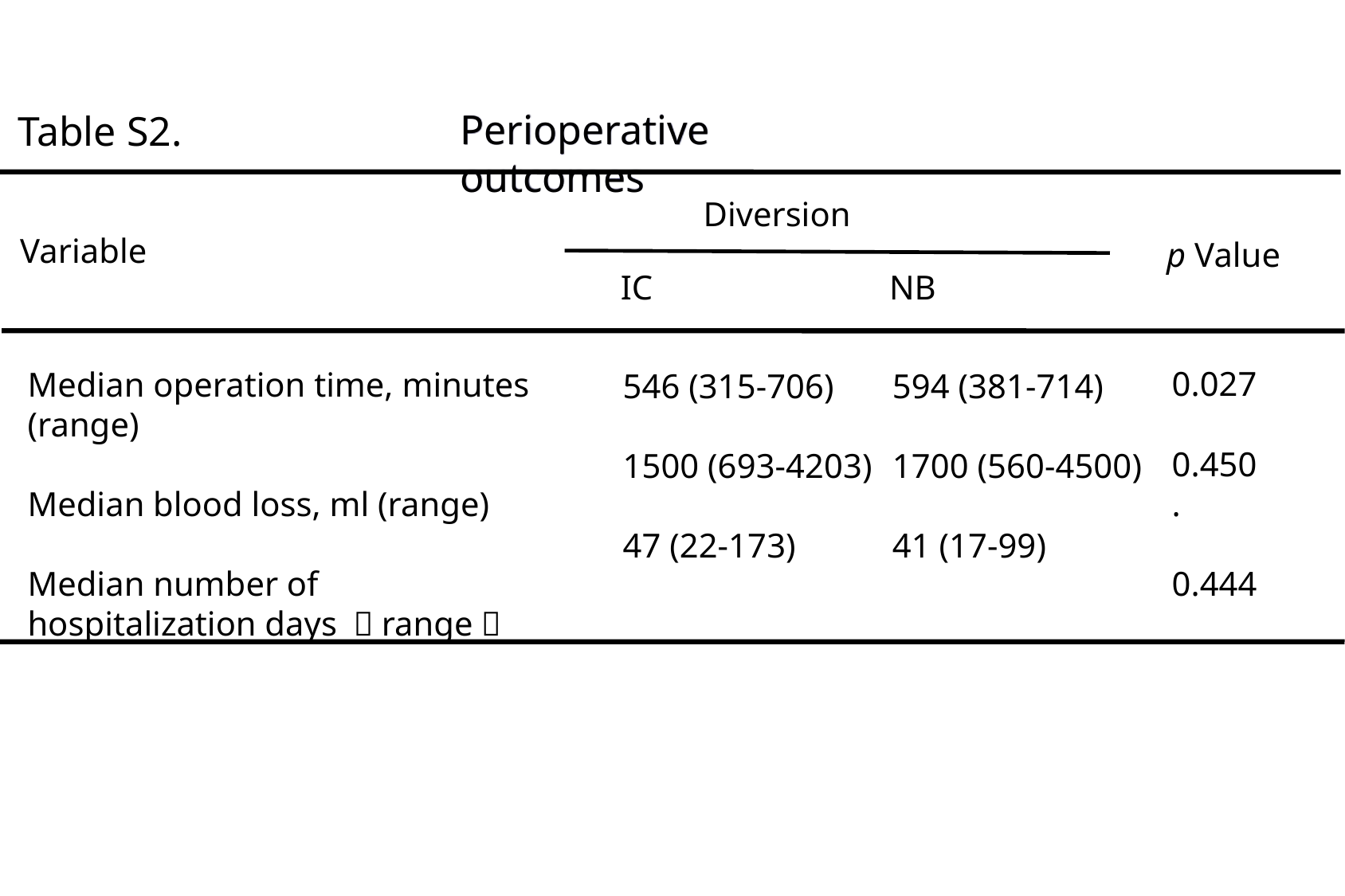

Perioperative outcomes
Table S2.
Diversion
Variable
p Value
IC
NB
0.027
0.450.
0.444
Median operation time, minutes (range)
Median blood loss, ml (range)
Median number of
hospitalization days （range）
546 (315-706)
1500 (693-4203)
47 (22-173)
594 (381-714)
1700 (560-4500)
41 (17-99)
