# Supplementary material for: Anterior urethra sparing cystoprostatectomy for bladder cancer: a 10-year, single center experience
Source: Springerplus. 2015 Aug 8;4:401. doi: 10.1186/s40064-015-1200-7 (PMC4529429; doi:10.1186/s40064-015-1200-7)
Supplement: Additional file 3: — Table S3. Perioperative complications. [file 40064_2015_1200_MOESM3_ESM.ppt]

## Slide 1
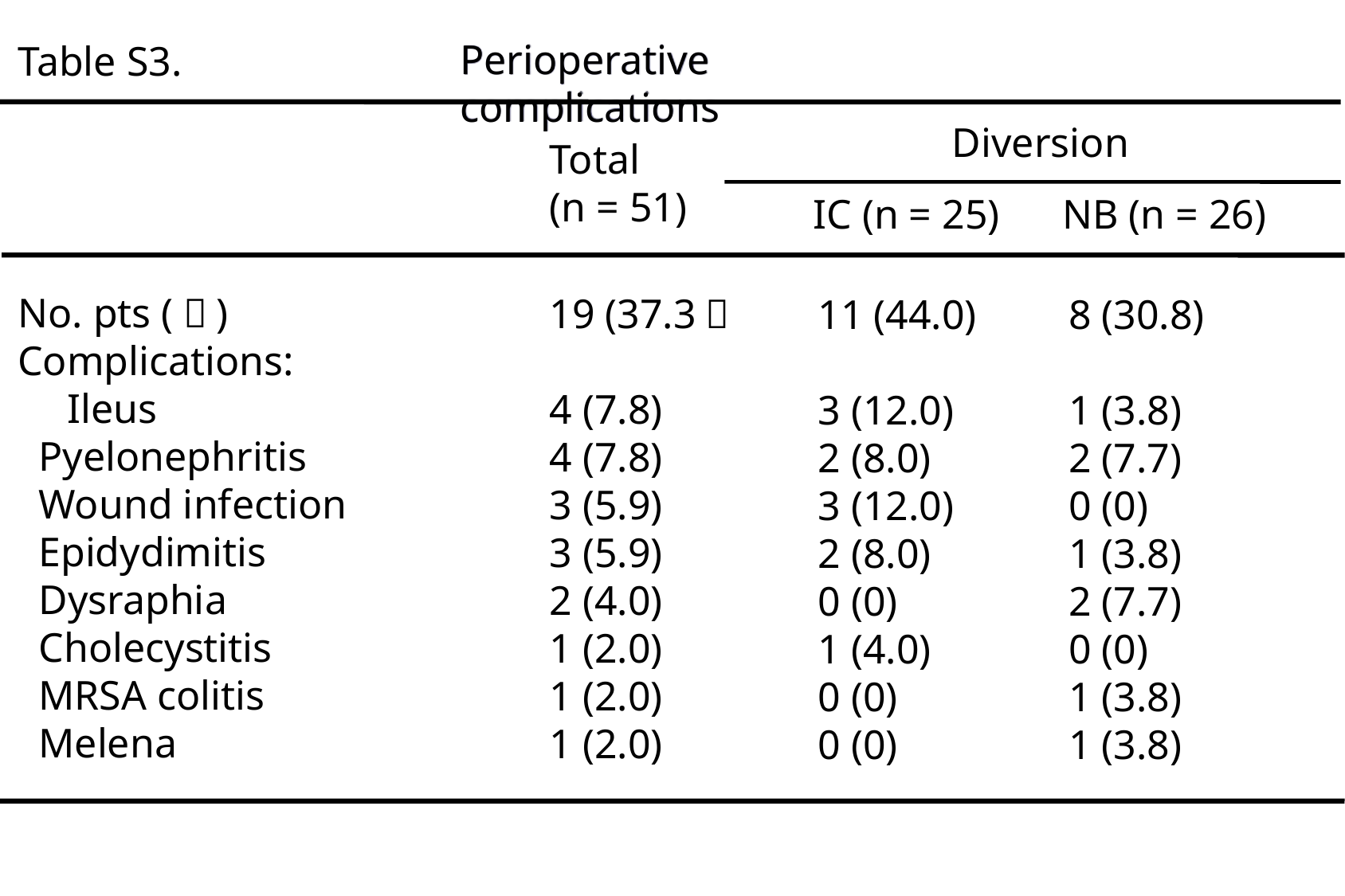

Perioperative complications
Table S3.
Diversion
IC (n = 25)
NB (n = 26)
No. pts (％)
Complications:
　Ileus
 Pyelonephritis
 Wound infection
 Epidydimitis
 Dysraphia
 Cholecystitis
 MRSA colitis
 Melena
19 (37.3）
4 (7.8)
4 (7.8)
3 (5.9)
3 (5.9)
2 (4.0)
1 (2.0)
1 (2.0)
1 (2.0)
11 (44.0)
3 (12.0)
2 (8.0)
3 (12.0)
2 (8.0)
0 (0)
1 (4.0)
0 (0)
0 (0)
8 (30.8)
1 (3.8)
2 (7.7)
0 (0)
1 (3.8)
2 (7.7)
0 (0)
1 (3.8)
1 (3.8)
Total
(n = 51)
