# Supplementary material for: Anterior urethra sparing cystoprostatectomy for bladder cancer: a 10-year, single center experience
Source: Springerplus. 2015 Aug 8;4:401. doi: 10.1186/s40064-015-1200-7 (PMC4529429; doi:10.1186/s40064-015-1200-7)
Supplement: Additional file 4: — Table S4. Multivariate analysis of factors associated with RFS and DSS. [file 40064_2015_1200_MOESM4_ESM.ppt]

## Slide 1
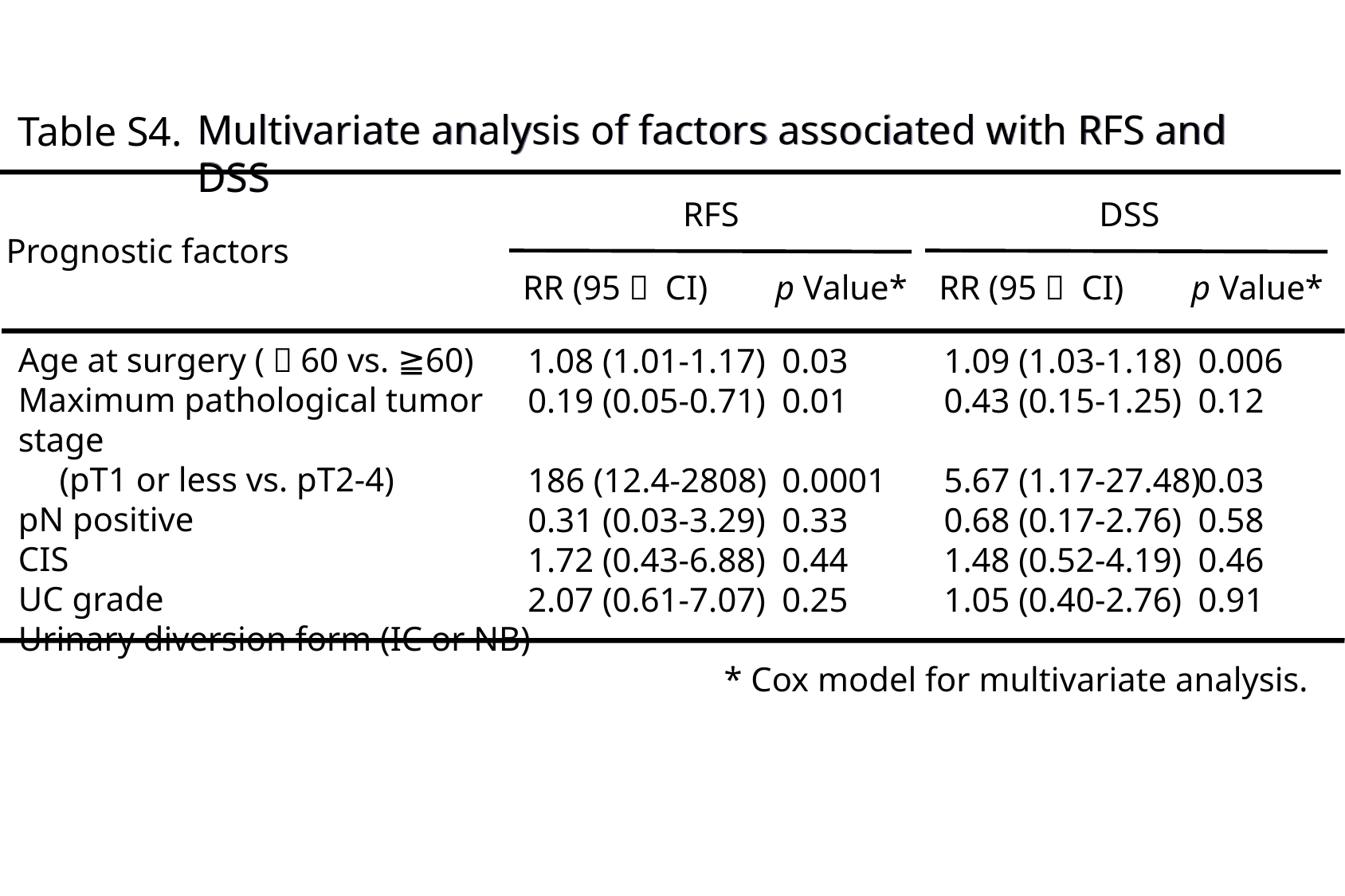

Multivariate analysis of factors associated with RFS and DSS
Table S4.
RFS
RR (95％ CI)
p Value*
1.08 (1.01-1.17)
0.19 (0.05-0.71)
186 (12.4-2808)
0.31 (0.03-3.29)
1.72 (0.43-6.88)
2.07 (0.61-7.07)
0.03
0.01
0.0001
0.33
0.44
0.25
DSS
RR (95％ CI)
p Value*
1.09 (1.03-1.18)
0.43 (0.15-1.25)
5.67 (1.17-27.48)
0.68 (0.17-2.76)
1.48 (0.52-4.19)
1.05 (0.40-2.76)
0.006
0.12
0.03
0.58
0.46
0.91
Prognostic factors
Age at surgery (＜60 vs. ≧60)
Maximum pathological tumor stage
　(pT1 or less vs. pT2-4)
pN positive
CIS
UC grade
Urinary diversion form (IC or NB)
* Cox model for multivariate analysis.
