# Supplementary material for: Anterior urethra sparing cystoprostatectomy for bladder cancer: a 10-year, single center experience
Source: Springerplus. 2015 Aug 8;4:401. doi: 10.1186/s40064-015-1200-7 (PMC4529429; doi:10.1186/s40064-015-1200-7)
Supplement: Additional file 5 — Table S5. Recurrence rates according to the site in patients with postoperative recurrence. [file 40064_2015_1200_MOESM5_ESM.ppt]

## Slide 1
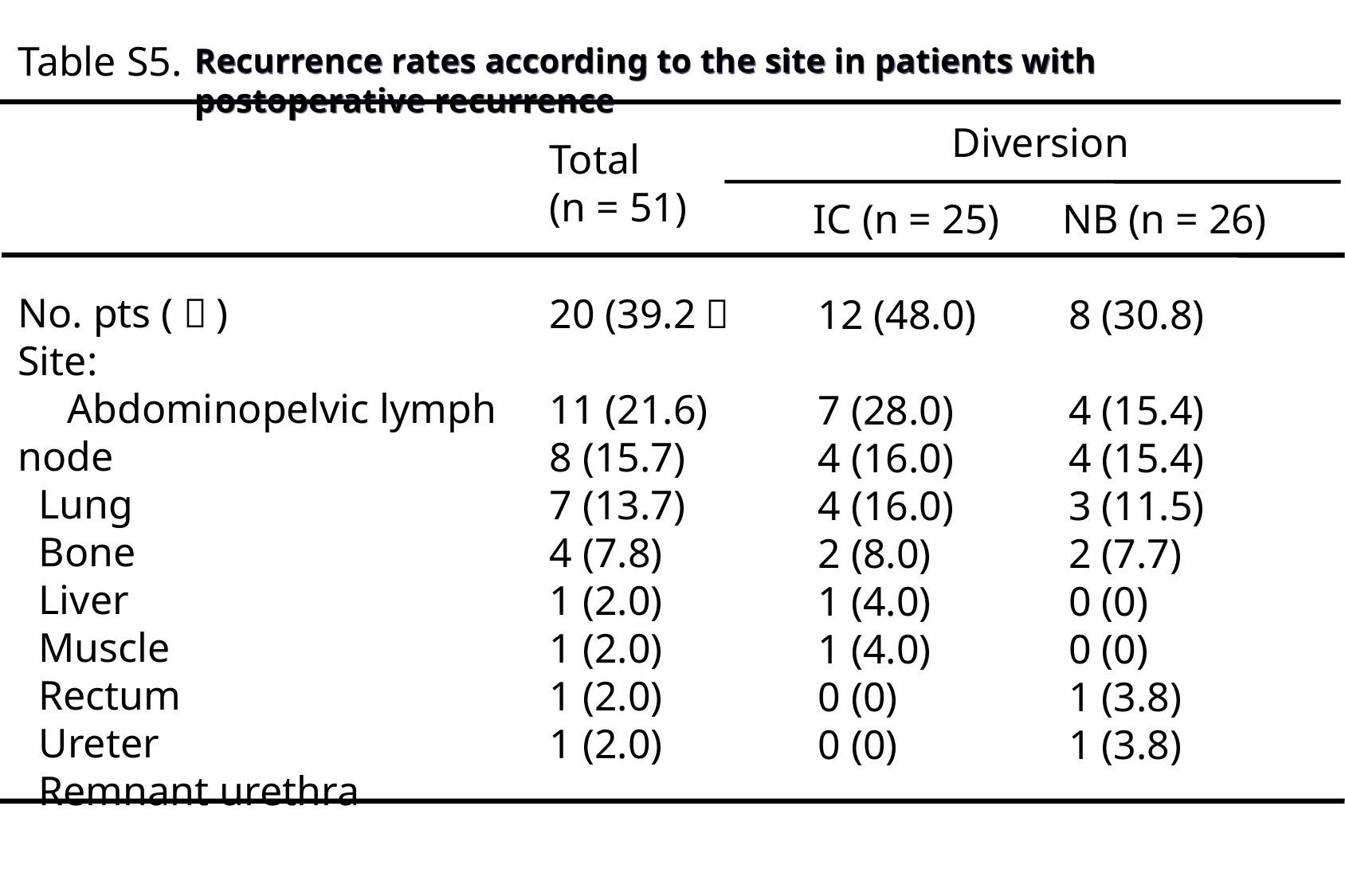

Table S5.
Recurrence rates according to the site in patients with postoperative recurrence
Diversion
Total
(n = 51)
IC (n = 25)
NB (n = 26)
No. pts (％)
Site:
　Abdominopelvic lymph node
 Lung
 Bone
 Liver
 Muscle
 Rectum
 Ureter
 Remnant urethra
20 (39.2）
11 (21.6)
8 (15.7)
7 (13.7)
4 (7.8)
1 (2.0)
1 (2.0)
1 (2.0)
1 (2.0)
12 (48.0)
7 (28.0)
4 (16.0)
4 (16.0)
2 (8.0)
1 (4.0)
1 (4.0)
0 (0)
0 (0)
8 (30.8)
4 (15.4)
4 (15.4)
3 (11.5)
2 (7.7)
0 (0)
0 (0)
1 (3.8)
1 (3.8)
